# The association between liver fibrosis and depression in patients after ischemic stroke

**DOI:** 10.1186/s12883-023-03091-z

**Published:** 2023-01-31

**Authors:** Yun Zhang, Yao Yang, Yue Feng, Zhonglun Chen, Lin He, Shu Xie, Jian Shi, Bufan Yang, Yufeng Tang

**Affiliations:** 1 Department of Psychosomatic Medicine, School of Medicine, Mianyang Central Hospital, University of Electronic Science and Technology of China, 12 Changjia Alley, Mianyang, 621000 Sichuan China; 2 Department of Clinical Laboratory, School of Medicine, Mianyang Central Hospital, University of Electronic Science and Technology of China, Mianyang, 621000 Sichuan China; 3 Department of Neurology, School of Medicine, Mianyang Central Hospital, University of Electronic Science and Technology of China, 12 Changjia Alley, Mianyang, 621000 Sichuan China

**Keywords:** Ischemic stroke, Depressive symptoms, Liver fibrosis, Fibrosis-4 index, Risk factor

## Abstract

**Background and objective:**

Liver fibrosis has been considered a predictor of cardiovascular disease. This study aimed to evaluate whether the degree of liver fibrosis is related to post-stroke depression (PSD) at 3 months follow-up.

**Methods:**

We prospectively and continuously enrolled patients with first-ever ischemic stroke from June 2020 to January 2022. Liver fibrosis was measured after admission by calculating the Fibrosis-4 index (FIB-4) and stratified into two categories (< 2.67 versus ≥ 2.67). Patients with a 17-item Hamilton Depression Scale score > 7 were further evaluated using the Chinese version of the structured clinical interview of DSM-IV, for diagnosing PSD at 3 months.

**Results:**

A total of 326 patients (mean age 66.6 years, 51.5% male) were recruited for the study. As determined by the FIB-4 score, 80 (24.5%) patients had advanced liver fibrosis. During the follow-up, PSD was observed in 91 patients, which accounted for 27.9% (95% confidence interval [CI] 25.5%–30.5%) of the cohort.

The prevalence of advanced liver fibrosis was higher in PSD patients than those without PSD (40.0% versus 24.0%; *P* = 0.006). After adjustment for covariates in the multivariate logistic analysis, advanced fibrosis was significantly associated with PSD (odds ratio [OR], 1.88; 95% CI, 1.03–3.42;* P* = 0.040). Similar results were found when the FIB-4 was analyzed as a continuous variable.

**Conclusions:**

This study found that advanced liver fibrosis was associated with an increased risk of 3-month PSD. FIB-4 score may be valuable for screening depressive symptoms in ischemic stroke patients.

## Introduction

Depression is one of the most common and serious neuropsychiatric sequelae after stroke, which accounts for approximately one-third of acute ischemic stroke survivors [1–3]. Post-stroke depression (PSD) is negatively associated with functional outcomes [4] and strongly related to an increased risk of mortality [5]. A meta-analysis of prospective studies with 4648 stroke patients further suggested that PSD may be an independent predictor of stroke recurrence [6]. Considering the clinical importance of PSD, the Guideline for Healthcare Professionals From the AHA/ASA recommended screening for PSD in stroke patients [7]. These results emphasize the urgency to early identify risk factors of PSD, which might have clinical implications for a better understanding of the etiology, early prevention, and intervention of PSD.

Non-alcoholic fatty liver disease (NAFLD) is estimated to affect approximately 25% of the adult population globally [8], and has recently been reported as a risk factor for large artery atherosclerosis and small vessel occlusion subtypes of stroke by mendelian randomization study [9]. The fibrosis stage is considered the most deleterious pathological feature of NAFLD [10]. A prospective stroke cohort showed that advanced liver fibrosis is associated with unfavorable long-term prognosis and stroke recurrence [11]. However, although liver fibrosis may not be rare in patients with ischemic stroke, there are few data available regarding the relationship between liver fibrosis and post-stroke affective disorder, especially PSD.

To date, several noninvasive modalities have been developed and established to evaluate the degree of liver fibrosis, including transient elastography and predictive scores [12–14]. Among these clinical scores, Fibrosis-4 (FIB-4) is easily obtained from blood test results and is recommended by guidelines to screen for liver fibrosis [15]. In this study, we evaluated the association between liver fibrosis, assessed by the FIB-4 index, and the development of PSD at 3 months in patients with ischemic stroke.

## Methods

### Study cohort and design

From June 2020 to January 2022, we performed a prospective study to enroll first-ever ischemic stroke patients with symptoms onset < 7 days in Mianyang Central Hospital. All patients met the diagnostic criteria for ischemic stroke according to the World Health Organization criteria [16]. The exclusion criteria were as follows: 1) age < 18 years old; 2) neurological deficits caused by other central nervous system diseases, such as cerebral hemorrhage, brain trauma, brain tumors, or paralysis after seizures; 3) history of dementia, cognitive dysfunction, depression or other psychiatric illness; 4) severe aphasia, dysarthria, understanding or consciousness disturbance that precluded us from performing the psychological evaluations.

### Baseline data assessment

Sociodemographic and clinical characteristics were recorded with a standard case report form after admission, including age, sex, education, body mass index, blood pressure, hypertension, diabetes mellitus, hyperlipidemia, and coronary heart disease. Stroke severity was measured using the National Institutes of Health Stroke Scale (NIHSS) score by trained neurologists at baseline [17]. Stroke etiology was confirmed by the Trial of Org 10,172 in Acute Stroke Treatment criteria including large-artery atherosclerosis, cardioembolism, small vessel disease, and others [18]. Laboratory tests were conducted within 24 h after admission (including platelet count, aspartate aminotransferase [AST], alanine aminotransferase [ALT], blood glucose, Hypersensitive C-reactive protein [Hs-CRP], and lipid profile). Biomarker levels were measured at our hospital’s laboratory by technicians who were blinded to the clinical data.

### Liver fibrosis measurement

The Fibrosis-4 (FIB-4) index was calculated to assess the degree of liver fibrosis for each participant after admission using the following formula: FIB-4 index = [age (years) × aspartate aminotransferase level (U/L)]/{[platelet count (10^9^/L)] × [alanine aminotransferase level (U/L)]1/2}. FIB-4 score is a well-validated and clinically established liver fibrosis index [11]. Based on the FIB-4 score, the severity of liver fibrosis was categorized into 2 groups: < 2.67, without advanced liver fibrosis; and ≥ 2.67, advanced liver fibrosis [19].

### Psychological measurement

In this study, we used the 17-item Hamilton Depression Rating Scale (17-HAMD) to assess the depressive symptoms at 3 months follow-up [20]. Patients with 17-HAMD score > 7 were further evaluated for diagnosing PSD according to the Structured Clinical Interview for the Diagnostic and Statistical Manual of Mental Disorders, 4th edition (DSM-IV) [21–23]. The psychological evaluations were performed by an experienced clinician who was blind to other clinical and laboratory data.

### Statistical analysis

According to the normality of data distribution, continuous variables were demonstrated as mean (standard deviation [SD]) or median (interquartile range [IQR]), Categorical variables were summarized as counts and proportions. The differences between the two groups were compared using the Student’s t–test or Mann–Whitney U test for continuous variables and the chi–squared test for categorical variables. Binary logistic regression analysis was employed to assess variables associated with the presence of PSD. All multivariable analyses were first adjusted for age and sex (Model 1) and additionally adjusted for all variables with* P* < 0.1 in univariate analysis (age, sex, education years, diabetes, baseline NIHSS score, and Hs-CRP levels; Model 2). We further evaluated the pattern and magnitude of the association of the FIB-4 index with PSD with restricted cubic splines with 4 knots (at fifth, 35th, 65th, and 95th percentiles) adjusted for the same potential confounders included in model 2 [24]. The results were expressed as an adjusted odds ratio (OR) with the corresponding 95% confidence interval (CI).

Furthermore, the net reclassification index and integrated discrimination improvement were calculated to estimate the predictive value of adding liver fibrosis status into models 1 and 2, separately. All statistical analysis was conducted with SPSS version 24.0 (SPSS Inc., Chicago, IL, USA) and R statistical software version 4.0.0 (R Foundation, Vienna, Austria), and a two-sided *P* value < 0.05 was considered to be statistically significant.

## Results

### Baseline characteristics

A total of 479 patients were screened from June 2020 to January 2022, with 326 patients eligible for the study. The average age of all patients was 66.6 ± 11.3 years and 168 (51.5%) were male. The median (IQR) NIHSS score on admission was 5.0 (2.0, 8.0).

### Advanced liver fibrosis in acute ischemic patients

Among these patients, 80 (24.5%) patients had advanced liver fibrosis. The demographic characteristics, clinical data, and laboratory data stratified by the degree of liver fibrosis were presented in Table 1. Compared to participants without liver fibrosis, those with advanced liver fibrosis were older (72.3 ± 8.4 years versus. 64.8 ± 11.5 years,* P* < 0.001), and had a higher prevalence of hypertension (82.5% versus. 70.4%,* P* = 0.038), cardioembolism (28.8% versus. 16.3%,* P* < 0.001) and 3-month PSD (40.0% versus. 24.0%,* P* = 0.006), and higher levels baseline NIHSS score (median, 6.0 versus. 5.0,* P* = 0.013).

**Table 1:** Baseline data of the included patients stratified by liver fibrosis status

Variables	Study cohort *n* = 326	With advanced liver fibrosis *n* = 80	Without advanced liver fibrosis *n* = 246	*P* value
Demographic characteristics				
Age, year	66.6 ± 11.3	72.3 ± 8.4	64.8 ± 11.5	< 0.001
Male, %	168 (51.5)	45 (56.3)	123 (50.0)	0.331
Education < 12 years, %	172 (52.8)	45 (56.3)	127 (51.6)	0.547
Risk factors, %				
Hypertension	240 (73.6)	66 (82.5)	174 (70.4)	0.038
Diabetes mellitus	83 (25.5)	22 (27.5)	61 (24.8)	0.630
Hyperlipidemia	69 (21.2)	20 (25.0)	49 (19.9)	0.334
Coronary heart disease	46 (14.1)	15 (18.8)	31 (12.6)	0.170
Current smoking	137 (42.0)	39 (48.8)	98 (39.8)	0.161
Clinical data				
Systolic blood pressure, mmHg	136.1 ± 19.7	136.4 ± 25.7	136.2 ± 17.4	0.891
Diastolic blood pressure, mmHg	82.0 ± 9.9	81.2 ± 9.5	82.3 ± 10.0	0.910
Body mass index, kg/m^2^	24.3 ± 2.5	24.1 ± 2.3	24.3 ± 2.6	0.431
Baseline NIHSS, score	5.0 (2.0, 8.0)	6.0 (4.0, 8.0)	5.0 (2.0, 8.0)	0.013
PSD at 3-month, %	91 (27.9)	32 (40.0)	59 (24.0)	0.006
Stroke subtypes, %				< 0.001
arge artery atherosclerosis	149 (45.7)	25 (31.3)	124 (50.4)	
Cardioembolism	63 (19.3)	23 (28.8)	40 (16.3)	
Small vessel occlusion	91 (27.9)	19 (23.8)	72 (29.3)	
Others	23 (7.1)	13 (16.3)	10 (4.1)	
Side of infarction, %				
Left	165 (50.6)	43 (53.8)	122 (49.6)	0.518
Right	181 (55.5)	39 (48.8)	142 (57.7)	0.161
Laboratory data				
Platelet, 10^9^/L	183.8 ± 58.2	145.9 ± 39.0	196.1 ± 58.2	< 0.001
Aspartate transaminase, U/L	29.2 ± 19.2	41.0 ± 30.6	25.3 ± 11.3	< 0.001
Alanine transaminase, U/L	32.6 ± 20.4	34.4 ± 29.2	32.0 ± 16.5	0.363
Total cholesterol, mmol/L	4.0 ± 1.1	4.1 ± 1.0	4.0 ± 1.1	0.646
Triglyceride, mmol/L	1.3 (1.0, 1.8)	1.3 (1.0, 1.8)	1.3 (1.1, 1.8)	0.698
High-density lipoprotein, mmol/L	1.0 ± 0.2	1.0 ± 0.3	1.0 ± 0.2	0.830
Low-density lipoprotein, mmol/L	2.4 (1.8, 2.9)	2.4 (1.9, 3.0)	2.4 (1.7, 2.9)	0.374
Blood glucose, mmol/L	5.4 ± 1.6	5.3 ± 1.2	5.4 ± 1.8	0.465
Hs-CRP, mg/L	4.0 (1.0, 7.1)	4.0 (1.0, 7.0)	4.0 (1.4, 7.0)	0.751

*Hs-CRP* Hypersensitive C-reactive protein, *NIHSS* National Institutes of Health Stroke Scale, *PSD* post-stroke depression

### Association of advanced liver fibrosis with PSD

During the 3 months follow-up, 91 (27.9%) patients experienced PSD. Compared with non-PSD patients, the PSD group had a higher prevalence of education < 12 years (64.8% versus. 48.1%, *P* = 0.007), diabetes mellitus (44.0% versus. 18.3%,* P* < 0.001), and advanced liver fibrosis (35.2% versus. 20.4%,* P* = 0.006), and had a higher level of FIB-4 score (median, 2.3 versus. 1.8,* P* = 0.032), baseline NIHSS score (median, 5.0 versus. 5.0,* P* = 0.048), and Hs-CRP (median, 5.6 mg/L versus. 4.0 mg/L,* P* = 0.015) (Table 2).

**Table 2:** Comparison of baseline data between patients with and without 3-month PSD

Variables	With PSD (*n* = 91)	Without PSD (*n* = 235)	*P* value
Demographic characteristics,			
Age, year	68.1 ± 10.8	66.0 ± 11.5	0.126
Male, %	42 (46.2)	126 (53.6)	0.226
Education < 12 years, %	59 (64.8)	113 (48.1)	0.007
Risk factors, %			
Hypertension	69 (75.8)	171 (72.8)	0.547
Diabetes mellitus	40 (44.0)	43 (18.3)	< 0.001
Hyperlipidemia	14 (15.4)	55 (23.4)	0.112
Coronary heart disease	16 (17.6)	30 (12.8)	0.262
Current smoking	34 (37.4)	103 (43.8)	0.289
Clinical data			
Systolic blood pressure, mmHg	136.9 ± 24.9	136.0 ± 17.4	0.640
Diastolic blood pressure, mmHg	82.4 ± 9.5	82.0 ± 10.1	0.677
Body mass index, kg/m^2^	24.6 ± 2.7	24.2 ± 2.4	0.198
Baseline NIHSS, score	5.0 (3.0, 8.0)	5.0 (2.0, 8.0)	0.048
Advanced liver fibrosis, %	32 (35.2)	48 (20.4)	0.006
FIB-4 index	2.3 (1.3, 3.1)	1.8 (1.3, 2.5)	0.032
Stroke subtypes, %			0.321
Large artery atherosclerosis	42 (46.2)	107 (45.5)	
Cardioembolism	20 (22.0)	43 (18.3)	
Small vessel occlusion	20 (22.0)	71 (30.2)	
Others	9 (9.9)	14 (6.0)	
Side of infarction, %			
Left	46 (50.5)	119 (50.6)	0.989
Right	53 (58.2)	128 (54.5)	0.539
Laboratory data			
Platelet, 10^9^/L	177.5 ± 60.2	186.2 ± 57.4	0.225
Aspartate transaminase, U/L	31.8 ± 24.2	28.2 ± 16.9	0.135
Alanine transaminase, U/L	34.0 ± 21.3	32.0 ± 20.0	0.429
Total cholesterol, mmol/L	4.0 ± 1.1	4.0 ± 1.1	0.987
Triglyceride, mmol/L	1.4 (1.1, 1.8)	1.3 (1.0, 1.8)	0.392
High-density lipoprotein, mmol/L	1.0 ± 0.2	1.0 ± 0.2	0.228
Low-density lipoprotein, mmol/L	2.5 (1.9, 3.0)	2.3 (1.8, 2.9)	0.311
Blood glucose, mmol/L	5.3 ± 1.7	5.4 ± 1.6	0.543
Hs-CRP, mg/L	5.6 (2.0, 7.0)	4.0 (1.0, 6.0)	0.015

*NIHSS* National Institutes of Health Stroke Scale, *PSD* post-stroke depression

**Fig. 1:**
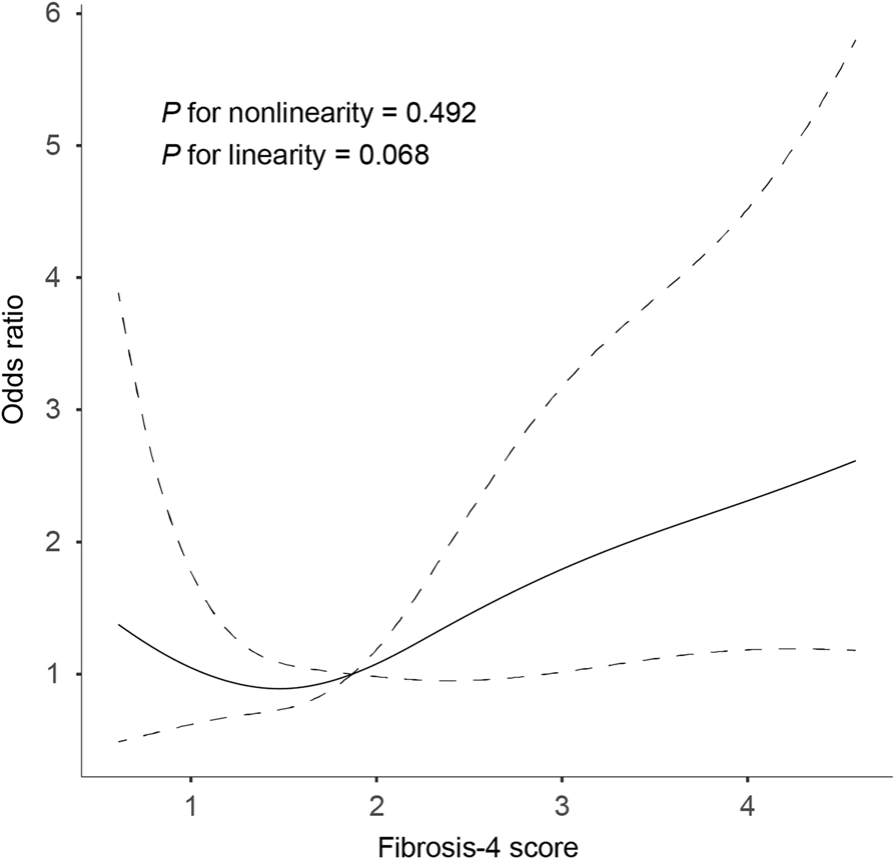
Association of fibrosis-4 (FIB-4) index with odds ratios of post-stroke depression (PSD) at 3 months. The restricted cubic spline of odds ratios and 95% confidence intervals with 4 knots located at the 5th, 35th, 65th, and 95th percentiles of the distribution of the FIB-4 score. The midpoints of FIB-4 score reference groups from categorical analyses serve as the reference point. Odds ratios were controlled for the same covariates included in model 2 in Table 3

Table 3 illustrated the results of multivariate analysis for the association between the degree of liver fibrosis and PSD. After adjusting for potential confounders (including age, sex, education years, diabetes mellitus, baseline NIHSS score, and Hs-CRP levels), multivariable logistic regression analysis demonstrated that advanced liver fibrosis was independently associated with a higher risk of PSD (OR, 1.88; 95% CI, 1.03–3.42, *P* = 0.040). The observed association remained significant when the FIB-4 index was analyzed as a continuous variable (OR, 1.30; 95% CI, 1.02–1.66, *P* = 0.037). The multivariable spline regression model further confirmed a linear association between the FIB-4 score and PSD risk (*P* for linearity = 0.068, Fig. 1).

**Table 3:** Multivariate analysis according to the degree of liver fibrosis for PSD

Variables	Crude model		Model 1		Model 2	
	OR (95%CI)	*P* value	OR (95%CI)	*P* value	OR (95%CI)	*P* value
Parameters as continuous variables						
FIB-4 index	1.32 (1.07–1.62)	0.009	1.30 (1.03–1.63)	0.027	1.30 (1.02–1.66)	0.037
Parameters as categorical variables						
With advanced liver fibrosis	2.11 (1.24–3.61)	0.006	2.03 (1.15–3.55)	0.014	1.88 (1.03–3.42)	0.040

Model 1 adjusted for age and sex

Model 2 adjusted for age, sex, education years, diabetes, baseline NIHSS score and Hypersensitive C-reactive protein levels

*CL* Confidence interval, *OR* Odd ratio, *PSD* Post-stroke depression

Adding the FIB-4 score to a model containing conventional risk factors significantly improved risk reclassification for PSD (continuous net reclassification improvement, 0.326; 95% CI, 0.087 − 0.565, *P* = 0.008; integrated discrimination improvement, 0.053; 95% CI, 0.027 − 0.080, *P* < 0.001) (Table 4).

**Table 4:** Reclassification Statistics (95% CI) for PSD by liver fibrosis

Models	cNRI		IDI	
	Estimate (95% CI)	*P* value	Estimate (95% CI)	*P* value
Model 1				
+ FIB-4 index	0.293 (0.053 − 0.532)	0.017	0.017 (0.001 − 0.034)	0.039
+ Advanced liver fibrosis	0.262 (0.035 − 0.489)	0.024	0.021 (0.004 − 0.036)	0.016
Model 2				
+ FIB-4 index	0.326 (0.087 − 0.565)	0.008	0.053 (0.027 − 0.080)	< 0.001
+ Advanced liver fibrosis	0.526 (0.293 − 0.759)	< 0.001	0.056 (0.029 − 0.082)	< 0.001

Model 1 adjusted for age and sex

Model 2 adjusted for age, sex, education years, diabetes, baseline NIHSS score and Hypersensitive C-reactive protein levels

*CI* confidence interval, *cNRI* continuous net reclassification improvement, *IDI* integrated discrimination improvement, *PSD* Post-stroke depression

## Discussion

In recent decades, the extensive focus on PSD is fully justified because of its negative impact on quality of life, cognitive and functional performance, and mortality after stroke [1–3, 25]. We found that advanced liver fibrosis was associated with increased odds for depression at 90 days post-stroke, after adjustment for potential confounders including demographic characteristics and baseline stroke deficits. Furthermore, adding the severity of liver fibrosis into PSD risk stratification may improve case identification.

In our cohort, we observed that 27.9% of ischemic stroke patients were diagnosed with depression 3 months later, which was similar to a recent systematic review indicating that the frequency of PSD is 33.0% (95% CI, 29% to 36%) [26]. Cumulative evidence has shown that PSD is associated with several well-known risk factors such as demographic characteristics, medical history, and stroke characteristics [21, 27, 28]. PSD is highly prevalent among both men and women. However, Chen et al. found that PSD is more common in women than men [28]. As for stroke location, Robinson et al. suggested that patients with left hemisphere lesions are more likely to have PSD than those without it, which is supported by other scholars [29]. But Sun et al. indicated that there was a significant correlation between depressive symptoms and right hemisphere lesions 6 months after stroke [27]. Also, our study did not confirm the association of PSD with females and stroke location. The discrepancy in risk factors of PSD might be due to the different time points in performing the psychological measurement. These results further emphasize the need for an internationally agreed definition of PSD.

To date, several cross-sectional studies have evaluated liver function in predicting clinical outcomes in stroke patients [11, 30–32]. A single-center study enrolling Korean found that the risk of long-term mortality was increased in ischemic stroke patients with significant liver fibrosis evaluated by transient elastography [30]. Similar results were found when liver fibrosis was evaluated by FIB-score [11]. In addition, liver fibrosis was reported to be associated with the risk of hemorrhagic transformation in ischemic stroke patients without the clinically overt liver disease [31]. Another recent study recruited patients with largely normal liver chemistries and found that the FIB-4 score and the aspartate aminotransferase/platelet ratio index were associated with admission hematoma volume, hematoma expansion, and mortality after intracerebral hemorrhage [32]. Apart from the functional outcome, our study further confirmed the hypothesis that the development of depressive symptoms may mediate by the degree of liver fibrosis.

Although the exact mechanism by which liver fibrosis affects PSD after acute stroke is not fully elucidated, several explanations may account for the observed association. Firstly, the inflammatory response is commonly present in all stages of liver disease and related to the presence and development of fibrosis, cirrhosis, and hepatocellular carcinoma [33]. At present, inflammation has attracted considerable attention as an important mechanism of PSD. The activation of several pro-inflammatory cytokines might induce alternations in brain function including the alteration of neurotransmission and increasing activity of the HPA axis [34], which may contribute to an increased risk of PSD. Secondly, Jagavelu et al. found that liver fibrosis could cause endothelial dysfunction [35]. Meanwhile, endothelial dysfunction has been shown to regulate depressive symptoms in a general elderly population [36]. Furthermore, non-alcoholic fatty liver disease has been associated with several subclinical atherosclerosis markers including carotid intimal medial thickness carotid plaques [37]. According to the population-based PREVENCION study among South American Hispanics, depressive symptoms were associated with subclinical atherosclerosis in men [38]. These data demonstrated that liver fibrosis plays an important role in the pathobiology of PSD by mediating subclinical atherosclerosis.

Our study had several limitations that need to be addressed. Firstly, this study is a single-center design with a relatively homogeneous ischemic stroke sample, which may have inherent selection bias and limit the generalization to other populations. Secondly, the degree of liver fibrosis was tested only at baseline after stroke without serial measurements, we were unable to exclude the impact of stroke on liver fibrosis. Thirdly, our study excluded the ischemic stroke patients with aphasia, dysarthria, and severe neurological deficits that precluded us from performing the psychological evaluations, which could have resulted in an underestimation of actual PSD rates. Finally, due to the observational nature of this study, it cannot demonstrate a causal relationship. These issues should be resolved in further longitudinal cohort studies with large samples.

## Conclusions

In conclusion, this investigation suggested that the advanced liver fibrosis assessed by the FIB-4 score was significantly correlated to the development of PSD 3 months after stroke. Ischemic stroke patients should be monitored for liver fibrosis and followed-up with appropriate interventions. Also, these findings should be further ascertained by animal experiments and cohort studies using more representative hospital-based samples.

## Data Availability

The data that support the findings of this study are available on request from the corresponding author.
